# Menstrual disorders in amateur dancers

**DOI:** 10.1186/s12905-019-0779-1

**Published:** 2019-07-03

**Authors:** Joanna Witkoś, Piotr Wróbel

**Affiliations:** Faculty of Medicine and Health Science, Andrzej Frycz Modrzewski Krakow University, Krakow, G. Herlinga - Grudzińskiego Street 1, 30-705 Kraków, Poland

**Keywords:** Menstrual disorders, Dancers, Amateur

## Abstract

**Background:**

Rigorous exercise undertaken by young girls, combined with a negative energetic balance, is related to substantial physiological changes in a competitor’s body, often leading to hormonal imbalance manifested by: delayed puberty, delayed menstruation, menstrual disorders, and even long-term secondary amenorrhoea. Very lean competitors, with insufficient body fat content, are not capable of maintaining oestrogen production at an optimal level, which results in hypothalamic-pituitary-gonadal axis abnormalities and menstrual disorders. Studies involving research on menstrual disorders in amateur dancers are scarce. Therefore, the aim of the study was to investigate whether menstrual disorders are present among young amateur dancers.

**Methods:**

The study involved 233 females of a mean age of 22.1 years (SD ± 4.9), training amateur ballroom dancing. The questionnaire included questions regarding the regularity of the menstrual cycle, including the absence of a menstrual period, causes of menstrual disorders and breakthrough bleeding.

**Results:**

Menstrual disorders following a period of regular menstruation were reported by 132 (56.7%) of ballroom dancers. In 105 (79.5%), the absence of a menstrual period lasted for less than 3 months, in 18 (13.6%) it persisted for 3 up to 6 months, while in 9 (6.8%) it lasted for over 6 months.

**Conclusions:**

The research conducted concluded that with an increased number of hours spent in training by amateur dancers, there was a statistically significant increase in the chance for menstrual disorders; the more training sessions per week there were, the longer the breaks in menstruation were noted.

**Electronic supplementary material:**

The online version of this article (10.1186/s12905-019-0779-1) contains supplementary material, which is available to authorized users.

## Background

Dance is a form of artistic expression and an artistic discipline enabling to convey emotions through movement. It is a unique blend of beauty and sport, requiring daily, long training and undertaking significant physical effort. In order to achieve success and desired artistic results, a dancer begins training at a very young age and continues it for years, also during puberty [1, 2]. In dance, which is a form of art, the aesthetics and attractiveness of competitors’ bodies are of fundamental importance. A slim silhouette is an essential and demanded prerequisite, hence the dancers have to maintain an appropriate diet, which significantly influences their future health, especially in the case of women [3, 4]. Young women, in order to achieve and maintain a desired, low body weight, alter their eating habits. These alterations mainly mean a reduced consumption of particular products and failure to provide the body with a sufficient amount of energy [5, 6]. Most often, such diets are not consulted with a dietician and may become dangerous for health, especially once they lead to destructive behaviour or mental disorders (anorexia, bulimia) [7].

Rigorous exercise undertaken by young girls, combined with a negative energetic balance, is related to substantial physiological changes in a competitor’s body, often leading to hormonal imbalance manifested by: delayed puberty, delayed menstruation, menstrual disorders, and even long-term secondary amenorrhoea (cessation of regular menses for three months or the cessation of irregular menses for six months) [8]. According to Frisch’s theory, menarche is dependent on achieving a critical body weight and determined adipose tissue content in the body. Conforming to the above-mentioned theory, menarche follows the moment when adipose tissue constituted at least 17% of overall body weight; it has to exceed 22% in order to maintain regular menstrual cycles [9–11%]. Adipose tissue is crucial, as this is where the metabolism of androgens into oestrogen and oestrogens into 17-beta-estradiol take place. These hormones are responsible for the appropriate functioning of the hypothalamic-pituitary-gonadal (HPG) axis. Very lean competitors, therefore, with insufficient body fat content, are not capable of maintaining oestrogen production at an optimal level, which results in HPG axis abnormalities and menstrual disorders [9–11].

Competitive sport and intensive exercise might lead to the development of a number of symptoms which were described in 1992 as the “Female Athlete Triad syndrome” or the “Triad”. These terms denote a correlation between low energy supply, that is eating disorders, and amenorrhoea or menstrual disorders, as well as decreased bone density or diagnosed osteoporosis [12–14]. The female reproductive system is particularly sensitive to environmental factors, including, among others, current body weight, diet, stress, amounts of sleep and rest, as well as undertaken training, including excessive physical effort. These factors have a fundamental influence on the reproductive system, especially during puberty, when the HPG axis remains immature. Adding to the above-mentioned aspects elements such as genetic factors, physique, and energetic balance (especially negative), one can observe a major influence on the timing of menarche and irregularity of menstrual cycles [15–17].

During puberty, changes in the female body consist in increasing the activity of the HPG axis, which is initiated by the pulsatile secretion of gonadoliberin (GnRH) by the hypothalamus. As a consequence, the secretion of gonadotropins – that is luteinising hormone (LH) and follicle-stimulating hormone (FSH) – is stimulated by the anterior pituitary gland; the concentration of gonadal steroid hormones also increases [18, 19]. An increase in the intensity of physical effort is followed by the inhibition of the hypothalamus’ activity, as well as a decrease in the frequency and amplitude of LH secretion pulses. A luteal phase defect occurs, which is characterised by the decrease of FSH production during the transformation period between luteal and follicular phases. Moreover, a decrease in oestradiol and progesterone secretion occurs in both phases, along with the shortening or complete disappearance of the luteal phase accompanied by the lengthening of the follicular phase. Secondary amenorrhoea in women undertaking extensive physical effort is therefore ascribed to the disorders of the hypothalamus-pituitary axis, connected with low levels of GnRH, gonadotropins and oestrogens. All the above-mentioned alterations in the female endocrine system may lead to anovulatory cycles and infertility among young female athletes [18–20]. Leptin, whose secretion is also inhibited during intensive physical effort, also has a considerable influence on puberty [21–23]. Special attention is also ascribed to the interactions between the HPG axis and testosterone, along with hyperandrogenism [24–26] connected with it, and thyroid hormones [27].

Studies involving research on menstrual disorders in amateur dancers are scarce. It remains unknown whether changes related to menstrual disorders affect equally professional dancers, including ballet dancers, and young amateur dancers, whose number is considerable; as sometimes they even outnumber the professional elites. Therefore, the aim of this study was to investigate whether menstrual cycle disorders also occur in young females who dance as amateurs.

## Methods

The study involved 233 females of a mean age of 22.1 years (SD ± 4.9), training amateur ballroom dancing. The dancers had trained, on average, for 6.2 ± 3.8 years, and they began training at a mean age of 16 ± 1.1 years. The number of training sessions per week amounted to 4 ± 2.8, while the number of hours per one training amounted to 1.9 ± 1. Dancers’ height was 165 ± 0.07 cm, and body weight 56.4 ± 7.9 kg. Considering the Body Mass Index (BMI), in the whole studied group, it was proven that on average it amounted to 20.7 ± 2.9, yet BMI below 18.5, denoting underweight, was revealed in 46 (19.7%) of investigated dancers and it amounted to 17.3 ± 1.6. 10 women had BMI indicating overweight (26.3 ± 2), and this represented 4.3% of participants. 151 females declared to be on a weight reduction diet, which amounted to 64.8%. The dancers’ diet was mainly based on the reduction of food intake, and it was not consulted with a coach or dietician. The average age of menarche among surveyed dancers was 12.6 ± 1.3 years.

The control group consisted of 147 females, students of Medical University of Silesia in Katowice, of a mean age of 21.5 years (SD ± 1.4) who did not train any sports in their entire life. They have been chosen for the research as they were of a similar age to women from the study group. The same inclusion and exclusion criteria were adopted for both groups. In this group, the women’s mean height was 167 ± 5.8 cm, and their mean body weight was 63.2 ± 11.3 kg. Considering the BMI, in the control group, it was shown that it amounted to 22.7 ± 3.8 on average. 9 (6.1%) women had a BMI indicating underweight, of 17.3 ± 1.1, 13 (8.8%) women had a BMI indicating overweight, (26.9 ± 1.4), and 8 (5.4%) respondents had a BMI indicating obesity (33 ± 1.7). Women from the control group did not implement any dietary restrictions. The women’s mean age at menarche was 12.7 ± 1.3 years.

The inclusion criteria consisted in full and correct completion of a survey, experience of menarche, as well as refraining from the use of contraceptives. The exclusion criteria were as follows: lack of menarche, using hormonal contraception. A proprietary survey was used to conduct research. The questionnaire (Additional file 1) included questions regarding the regularity of the menstrual cycle, including amenorrhoea, causes of menstrual disorders and breakthrough bleeding.

Statistical analysis was performed with the use of logistic regression. Differences at the significance level of *p* < 0.05 were considered to be statistically significant. Additionally, a fraction test was performed to compare the percentage of women with menstrual disorders in the group of dancers versus the control group.

## Results

Research results showed that in 179 (76.8%) of dancers the general profile of menstrual cycles was regular, with bleeding occurring every 24 to 34 days (minimum 24.9, maximum 30.7, on average every 27.6 days). However, in 54 (22.2%) participants their menstrual cycles did not remain within the specified range; they were significantly shorter or longer, hence they were considered to be irregular cycles. The dancers admitted that 41 (17.6%) of them experienced breakthrough bleeding, yet were unable to explain its causes as they have not consulted the issue with a gynaecologist.

Among the dancers subject to assessment, 132 (56.7%) females reported the absence of a menstrual period which occurred at the moment of undertaking intensive physical training. Table 1 presents the number of months with the absence of menstruation and the number of women affected by this problem. The majority of dancers (37.1%) did not know the causes underlying the occurrence of their menstrual disorders; they also did not consult them with a gynaecologist. The possible causes mentioned by the dancers included: training and completion-related stress – 37.1% of women, followed by a reduction in body weight as a consequence of training and dietary restrictions - 15.9% of respondents, and hormonal disorders – 9.9% indications.

**Table 1:** Menstrual disorders occurring in surveyed dancers following a period of regular menstrual cycles

normal menstrual cycles	the absence of a menstrual period	less than 3 months	between 3 and 6 months	over 6 months
101 (43.3)	132 (56.7%)	105 (79.5%)	18 (13.6%)	9 (6.9%)

A statistical analysis of the obtained results has shown a significant correlation between the number of training sessions per week undertaken by surveyed dancers and a higher chance of menstrual disorders, namely amenorrhoea (Table 2).

**Table 2:** The correlation between the occurrence of menstrual disorders and years of training, number of training sessions per week and current body weight

Predictors	B	Wald test	*p*	OR	95% PU for OR	
					Bottom	Top
Years of training	.02	0.26	.610	1.02	.95	1.10
Training sessions per week	.14	6.56	.010	1.15	1.03	1.28
Body weight	−.02	1.9	.168	0.98	.94	1.01
Constant	.95	0.81	.367	2.59		

Nagelkerke’s *R*^*2*^*:* 0.06

Furthermore, a statistically significant correlation between the number of training sessions per week and the length of break in regular menstruation was determined. It has been shown that the more training sessions per week were undertaken by the investigated dancers, the longer the breaks in normal menstruation reported (Table 3).

**Table 3:** The correlation between the length of break in regular menstruation and years of training, the number of training sessions per week and current body weight

Predictors	*B*	*Beta*	*r*_*semi*_	variance %	*t*	*p*
Constant	1.36				3.70	< .001
Years of training	.01	.07	.06	.40	.99	.321
Training sessions per week	.04	.15	.14	1.94	2.18	.030
Body weight	.00	.02	.02	.02	.25	.806

*B*: partial regression coefficient

*Beta*: partial standardised regression coefficient

*r*_*semi*:_ semi-partial correlation coefficient

% variance: percent of variance of dependent variable, explained by a given predictor (calculated as squared semi-partial correlation multiplied by 100%)

*t*: value of Student’s *t* statistics

*p:* probability level

No statistical correlations were found between the occurrence of menstrual bleeding and number of training sessions and their intensity per week (Table 4). Only 12 (5.2%) girls knew the meaning of the term “triade” while only 9 (4%) underwent a bone density test. As many as 192 (82%) of the dancers gave a “no” answer to the following question: “Do you think that sport can – in any way – influence your future fertility and ability to conceive?”

**Table 4:** The correlation between the occurrence of breakthrough bleedings and years of training and their intensity in a week

Predictors	B	Wald test	*p*	OR	95% PU for OR	
					Bottom	Top
Years of training	.05	1.11	.292	1.05	.96	1.15
Number of training sessions per week	.10	2.85	.091	1.10	.98	1.24
Constant	−2.32	32.36	< .001	.10		

Nagelkerke’s *R*^*2:*^ 0.04

When the group of studied dancers was compared with the control group, it was found that the percentage of women reporting menstruation disorders was significantly higher in the group of dancers than in the control group of women who did not train any sports (*p* < 0.000001). No differences were found between the studied groups concerning the age of menarche.

## Discussion

The menstrual cycle is a complex physiological process which might be severely disrupted when a young female undertakes excessive physical effort. Sports training significantly increases the prevalence of menstrual cycle disorders in women; the earlier they start training, the higher the percentage of young women battling menstrual disorders. This percentage can amount up to 44% in females participating in competitive sports, while in sport disciplines requiring a thin silhouette, e.g. dancers, the frequency of menstrual disorders might amount to 75–79% [28].

Castelo-Branco and colleagues’ [29] paper indicated numerous factors which can influence menstrual disorders in sportswomen. The following were enumerated: type of sports discipline, intensity of training, number of training sessions per week, low energetic balance, low body mass index, body fat percentage, pathological eating habits and mental stress related to competitions. It was also determined that the start of intense physical training by women who formerly had a sedentary lifestyle causes – in some of them – menstrual disorders. Castelo-Branco and colleagues’ research [29] compared young dancers and young women that do not engage in any physical activity. A statistically significant difference was determined in both menarche delay and more frequent amenorrhoea in comparison with the control group. Dancers’ BMI was lower than that of the control group, and 32% of women followed weight-loss diets in comparison with 12% of females from the control group.

Menstrual disorders in females engaging in sports might also be a result of increased energy expenditure occurring as one undertakes physical effort not compensated by appropriate nutrition. The frequency of eating disorders is particularly noticeable among women engaging in a sport which requires one to have a slim figure. It is therefore concluded that it is not only increased physical activity that constitutes a possible cause of menstrual disorders, but chronic malnutrition, significant reduction of calorie intake and negative energetic balance which does not compensate raised energetic expenditure [3, 15]. Stokić et al. [3] evaluated the relation between menstrual disorders and adipose tissue mass in ballet dancers and a control group. According to the BMI, as much as 50% of dancers were underweight, in comparison to 23.3% of participants in the control group. Moreover, 66.7% of the dancers had a lower percentage of body fat. The following were also found: amenorrhoea in 20% of dancers, menarche delay in comparison with the control group, and longer menstrual cycles in comparison with the control group. Doyle-Lucas et al. [30] emphasise in their research that competitive dancing and ballet are definitely linked to an increased prevalence of the “Athlete Triad” among surveyed dancers. The research compared physical and behavioural characteristics of dancers and non-dancers. Irregular menstrual cycles were reported by 6 out of 15 dancers, while in the control group it was only 1 female out of 15 [30].The occurrence of the “triad” is inevitably linked to the risk constituted by impaired bone density and non-achievement of peak bone mass during puberty, leading in consequence to an increased risk of fractures, even at a young age, and especially following the menopause. In Keen and colleagues’ [31] research it was determined that even a few years of regular menstruation in women who used to train professionally and had menstrual disorders was not enough to ensure that their bone density remains the same as in women who menstruate uninterruptedly on a regular basis.

In conducted own research it was noted that 56.7% of amateur dancers suffered from amenorrhoea. Amenorrhoea (absence of menstruation following a period of regular bleeding) lasting for less than 3 months was reported by 79.5%; amenorrhoea lasting between 3 and 6 months was reported by 13.6%, while amenorrhoea lasting for over 6 months was reported by 6.8% of competitors. A statistical analysis indicated that the statistical chance for menstrual disorders increased along with greater number of hours spent in training. It was also determined that the more training sessions per week there were, the longer the breaks in menstruation were noted. On the other hand, on the basis of the analysis of correlation between the years of training, number of training sessions per week and amenorrhoea it was determined that the number of training sessions per week was a positive predictor value for amenorrhoea.

The age of menarche is one of the genetically conditioned biological characteristics. In the majority of women with a correct and regular diet, living in highly developed countries, and having an appropriate standard of living, including hygiene, social, and economic conditions, the first period usually occurs at the age of 12.5–12.8 years [32]. The age of menarche among the investigated dancers was, on average, 12.6 ± 1.3 years, hence it was not fundamentally different from the norm present in the population. Still, numerous scientific research studies have proven that females engaging in competitive sports start menstruating later than girls who refrain from increased physical activity. In many cases, therefore, menarche delay is caused by increased physical effort [32]. The results obtained show that menstrual disorders occur not only among females regularly engaging in competitive sport or ballet, but also in amateur dancers, and constitute a consequence of undertaking excessively intensive, exhausting and long-lasting physical effort as well as following weight-loss diets, which result in competitors’ malnutrition.

Irregular menstrual cycles are a common phenomenon in the first few years from menarche [33, 34]. The studied dancers were 22.1 ± 4.9 years old, on average, and they began training at a mean age of 16 ± 1.1 years, that is, about 4 years after menarche. Considering the above, it can be assumed that their menstrual disorders were related to regular dance trainings and physical efforts required by this sport. Differences in the body weight between the group of dancers and the control group should also be noted. Women in both groups were almost of the same age, of about 22 years; however, the mean body weight was 56.4 ± 7.9 kg for the dancers, and 63.2 ± 11.3 kg for women in the control group. These differences were certainly associated with regular physical trainings undertaken by the dancers and the dietary restrictions they imposed on themselves. Therefore, it can be said that a drop in body weight could also contribute to menstrual disorders in the dancers.

In the conducted survey, the question concerning menstrual cycle irregularities was constructed in such way that it also provided an answer to a question whether these women had correct monthly menstruation cycles before they started to train, and the menstruation stopped only when they started to train regularly. This question was: “Have you ever experienced a situation when your menstruation did not occur after a period of regular menstruations?” The majority of dancers who answered ‘yes’ to this question saw themselves a link between their menstrual disorders and regular physical trainings resulting in more intense physical exercise and a loss in the body weight.

Dusek [18] conducted an assessment of the influence of intense physical training on menstrual cycles in women aged 15–21. These women trained various sports disciplines; the following were included: volleyball and basketball players, ballet dancers and runners. The author demonstrated that the prevalence of secondary amenorrhoea was three times higher among investigated sportswomen than in the control group. Moreover, he also stated that the prevalence of primary amenorrhoea was statistically significantly higher in sportswomen that in the control group. The moment of menarche was also significantly delayed in sportswomen who started their sporting activity prior to their first period. The author of the paper concluded clearly that high-intensity physical training started prior to menarche significantly influences its delay. Hincapié and Cassidy’s [6] research analysed the prevalence of eating and menstrual disorders in professional dancers in comparison to amateur dancers. It was noted that 50% of professional dancers suffered from the above-mentioned issues, while among amateur dancers, a significantly smaller number of females was affected: from 13.6 to 26.5%. It was also noted that 32% of amateur dancers had menstrual disorders in the first year following the start of intense training. Bacchi et al. [2] also compared the occurrence of increased prevalence of menstrual disorders in amateur and professional dancers. It was shown that BMI was lower in both professional and amateur dancers, while the prevalence of menstrual disorders amounted to 51% among professional dancers in comparison with 34% of amateur dancers. The research showed that low body weight and menstrual disorders are also frequent phenomena among amateur dancers. Despite numerous research studies indisputably confirming the negative influence of intensive physical training and weight-loss diets on menstrual disorders, positive conclusions obtained by Lagowska et al. [35] should also be noted. It was determined that the return to correct nutrition in sportswomen and ballet dancer suffering from menstrual disorders might restore regular menstrual cycles, even though the regaining period might take longer than 1 year. It was noted that the increase of body fat mass might constitute one of the most important predictive factors in restoring menstruation.

In conclusion, to sum up our own study, it should be noted that the females under assessment, practising amateur dancing, are not aware that the absence of regular periods and related oestrogen deficiency lasting for many months, leads to the reduction of peak bone mass. Moreover, most of them are also unaware that intensified physical effort and associated hormonal changes may result in anovulatory cycles and related infertility. Unfortunately, for many young females engaged in sport activities, amenorrhoea constitutes a desired situation which enables them to train intensively throughout the month, as well as achieve better results at competitions.

## Conclusions

The research conducted concluded that with an increased number of hours spent in training by amateur dancers, there was a statistically significant increase in the chance for menstrual disorders; the more training sessions per week there were, the longer the breaks in menstruation were noted.

## Additional file

Additional file 1: Author's questionnaire regarding menstrual disorders. (DOCX 43 kb)

## Data Availability

The datasets used and/or analysed during the current study are available from the corresponding author on reasonable request.

## Abbreviations

BMI: Body mass index
FSH: Follicle-stimulating hormone
GnRH: Gonadoliberin
HPG: Hypothalamic–pituitary–gonadal
LH: Luteinising hormone

## References

[1] Koutedakis Y, Jamurtas A (2004). The dancer as a performing athlete: physiological considerations. Sports Med.

[2] Bacchi E, Spiazzi G, Zendrini G, Bonin C, Moghetti P (2013). Low body weight and menstrual dysfunction are common findings in both elite and amateur ballet dancers. J Endocrinol Investig.

[3] Stokić E, Srdić B, Barak O (2005). Body mass index, body fat mass and the occurrence of amenorrhea in ballet dancers. Gynecol Endocrinol.

[4] De Souza MJ, Williams NI (2004). Physiological aspects and clinical sequelae of energy deficiency and hypoestrogenism in exercising women. Hum Reprod Update.

[5] Peric M, Zenic N, Sekulic D, Kondric M, Zaletel P (2016). Disordered eating, amenorrhea, and substance use and misuse among professional ballet dancers: preliminary analysis. Med Pr.

[6] Hincapié CA, Cassidy JD (2010). Disordered eating, menstrual disturbances, and low bone mineral density in dancers: a systematic review. Arch Phys Med Rehabil.

[7] Mendelsohn FA, Warren MP (2010). Anorexia, bulimia, and the female athlete triad: evaluation and management. Endocrinol Metab Clin N Am.

[8] Orio F, Muscogiuri G, Ascione A, Marciano F, Volpe A, La Sala G, Savastano S, Colao A, Palomba S (2013). Effects of physical exercise on the female reproductive system. Minerva Endocrinol.

[9] Frisch RE (1996). The right weight: body fat, menarche, and fertility. Nutrition..

[10] Frisch RE (1993). Critical fat. Science..

[11] Frisch RE (1990). The right weight: body fat, menarche and ovulation. Baillieres Clin Obstet Gynaecol.

[12] Hoch AZ, Papanek P, Szabo A, Widlansky ME, Schimke JE, Gutterman DD (2011). Association between the female athlete triad and endothelial dysfunction in dancers. Clin J Sport Med.

[13] Papanek PE (2003). The female athlete triad: an emerging role for physical therapy. J Orthop Sports Phys Ther.

[14] Warren MP, Shantha S (2000). The female athlete. Baillieres Best Pract Res Clin Endocrinol Metab.

[15] Warren MP, Goodman LR (2003). Exercise-induced endocrine pathologies. J Endocrinol Investig.

[16] Warren MP (2011). Endocrine manifestations of eating disorders. J Clin Endocrinol Metab.

[17] Warren MP, Perlroth NE (2001). The effects of intense exercise on the female reproductive system. J Endocrinol.

[18] Dusek T (2001). Influence of high intensity training on menstrual cycle disorders in athletes. Croat Med J.

[19] De Souza MJ, Van Heest J, Demers LM, Lasley BL (2003). Luteal phase deficiency in recreational runners: evidence for a hypometabolic state. J Clin Endocrinol Metab.

[20] Cadegiani FA, Kater CE (2017). Hormonal aspects of overtraining syndrome: a systematic review. BMC Sports Sci Med Rehabil.

[21] Muñoz MT, de la Piedra C, Barrios V, Garrido G, Argente J (2004). Changes in bone density and bone markers in rhythmic gymnasts and ballet dancers: implications for puberty and leptin levels. Eur J Endocrinol.

[22] Kaufman BA, Warren MP, Dominguez JE, Wang J, Heymsfield SB, Pierson RN (2002). Bone density and amenorrhea in ballet dancers are related to a decreased resting metabolic rate and lower leptin levels. J Clin Encocrinol Metab.

[23] Baylor LS, Hackney AC (2003). Resting thyroid and leptin hormone changes in women following intense, prolonged exercise training. Eur J Appl Physiol.

[24] Łagowska K, Kapczuk K (2016). Testosterone concentrations in female athletes and ballet dancers with menstrual disorders. Eur J Sport Sci.

[25] Hagmar M, Berglund B, Brismar K, Hirschberg AL (2009). Hyperandrogenism may explain reproductive dysfunction in olympic athletes. Med Sci Sports Exerc.

[26] Javed A, Kashyap R, Lteif AN (2015). Hyperandrogenism in female athletes with functional hypothalamic amenorrhea: a distinct phenotype. Int J Women's Health.

[27] Nicoll JX, Hatfield DL, Melanson KJ, Nasin CS (2018). Thyroid hormones and commonly cited symptoms of overtraining in collegiate female endurance runners. Eur J Appl Physiol.

[28] Bale P, Doust J, Dawson D (1996). Gymnasts, distance, runners, anorexics body composition and menstrual status. J Sports Med Phys Fitness.

[29] Castelo-Branco C, Reina F, Montivero AD, Colodrón M, Vanrell JA (2006). Influence of high-intensity training and of dietetic and anthropometric factors on menstrual cycle disorders in ballet dancers. Gynecol Endocrinol.

[30] Doyle-Lucas AF, Akers JD, Davy BM (2010). Energetic efficiency, menstrual irregularity, and bone mineral density in elite professional female ballet dancers. J Dance Med Sci.

[31] Keen AD, Drinkwater BL (1997). Irreversible bone loss in former amenorrheic athletes. Osteoporos Int.

[32] Thomas F, Renaud F, Benefice E, de Meeüs T, Guegan JF (2001). International variability of ages at menarche and menopause: patterns and main determinants. Hum Biol.

[33] Adams Hillard PJ (2002). Menstruation in young girls: a clinical perspective. Obstet Gynecol.

[34] Diaz A, Laufer MR, Breech LL (2006). Menstruation in girls and adolescents: using the menstrual cycle as a vital sign. Pediatrics..

[35] Łagowska K, Kapczuk K, Jeszka J (2014). Nine-month nutritional intervention improves restoration of menses in young female athletes and ballet dancers. J Int Soc Sports Nutr.

