# Supplementary material for: Menstrual disorders in amateur dancers
Source: BMC Womens Health. 2019 Jul 3;19:87. doi: 10.1186/s12905-019-0779-1 (PMC6609389; doi:10.1186/s12905-019-0779-1)
Supplement: Supplementary file 1 — Author's questionnaire regarding menstrual disorders. (DOCX 43 kb) [file 12905_2019_779_MOESM1_ESM.docx]

The questionnaire

Date of birth:

Height:

Body weight:

Sports discipline:

Club name:

How many trainings are there every week?

How many hours does one training last?

For how many years have you been training?

| A | YES |  |
| --- | --- | --- |
| B | NO |  |

Do you menstruate:

State the age when the menstruation began ...............................................................................

| A | YES |  |
| --- | --- | --- |
| B | NO |  |

Are your periods regular, i.e. do they occur every 24–34 days?

How many days are there between your periods: minimum .........maximum .........average

| A | YES |  |
| --- | --- | --- |
| B | NO |  |

Does intermenstrual bleeding occur?

How long does it last for? ......................................................................................................

Did it happen that the period did not occur for a prolonged time following regular menstruation?

| A | YES |  |
| --- | --- | --- |
| B | NO |  |

| How long was the break in regular menstruation? | | YES | NO |
| --- | --- | --- | --- |
| A | less than 3 months |  |  |
| B | 3–6 months |  |  |
| C | over 6 months |  |  |

| What caused these disorders? | | YES | NO |
| --- | --- | --- | --- |
| A | endocrine disorders |  |  |
| B | stress |  |  |
| C | weight loss |  |  |
| D | unknown |  |  |
| E | other (what was it?) |  |  |

Has a doctor (gynaecologist) ever diagnosed you with endocrine disorders?

| A | YES (which?) |  |
| --- | --- | --- |
| B | NO |  |

Do you use hormonal contraception?

| A | YES (which?) |  |
| --- | --- | --- |
| B | NO |  |

Do you believe that sport can—in any way—influence your future fertility/ability to conceive?

| A  A | YES (why?) |  |
| --- | --- | --- |
| B | NO (why?) |  |

Do you know what Female Athlete Triad is?

| A | YES (according to you, what is it?) |  |
| --- | --- | --- |
| B | NO |  |

Have you ever had a bone density scan (bone densitometry)?

| A | YES |  |
| --- | --- | --- |
| B | NO |  |
